# Tryptophan supplementation and the response to unfairness in healthy volunteers

**DOI:** 10.3389/fpsyg.2015.01012

**Published:** 2015-07-16

**Authors:** Hilâl Cerit, Rachel J. Schuur, Ellen R. A. de Bruijn, Willem Van der Does

**Affiliations:** ^1^Clinical Psychology, Institute of Psychology, Leiden UniversityLeiden, Netherlands; ^2^Leiden Institute for Brain and CognitionLeiden, Netherlands; ^3^Department of Psychiatry, Leiden University Medical CenterLeiden, Netherlands

**Keywords:** serotonin, tryptophan supplementation, ultimatum game, unfairness, impulsivity

## Abstract

Experimental manipulation of serotonin (5-HT) availability has been shown to modulate social behavior. For instance, serotonin depletion increased the rejection rates of unfair offers in the ultimatum game (UG), whereas a single dose of the serotonin reuptake inhibitor (citalopram) decreased rejection rates. These effects were observed immediately after the manipulation. The aim of this study was to investigate the effect of prolonged tryptophan (TRP) supplementation on UG performance in healthy individuals. A randomized double-blind placebo (PLC)-controlled design was used. Healthy volunteers (*N* = 47) completed the UG before and after a 6-day intervention of TRP (2.8 g/day) or PLC. Impulsivity was measured with a Go-Stop task. The overall analyses showed that TRP supplementation had no significant effect on UG scores, but the direction of the effect was opposite from expectations. Because repeated performance of the UG may lead to unwanted learning effects or strategical changes, additional analyses were conducted in which participants (*N* = 7) who accepted all offers on the second measurement were excluded. These analyses revealed that the TRP-group rejected very unfair offers more often than the PLC group. The groups did not differ on impulsivity. Increasing serotonin through TRP supplements increased the rejection of very unfair offers. The direction of our findings is inconsistent with earlier studies that showed that increasing 5-HT availability results in less rejection of unfair offers. The current findings thus importantly suggest that effects of acute vs. prolonged enhancement of 5-HT availability may differ. Also, the outcomes show that the UG is a complex task and participants’ decisions may depend on context, e.g., prior experience with the task.

## Introduction

Substantial evidence from preclinical and clinical studies emphasize the importance of the serotonergic system in the regulation of social behavior (Sandi and Haller, 2015). Changes in the serotonergic system (e.g., receptor expression and serotonin levels) have repeatedly been reported to affect social behavior in animals and humans (Sandi and Haller, 2015). Specifically, low serotonin (5-HT) levels have not only been associated with aversive social behavior such as aggression (Brown et al., 1979), but also with psychopathological conditions such as depression (Owens and Nemeroff, 1994). Since 5-HT appears to play a key role in the regulation of social behavior and in maintaining mental health, studies have been conducted in order to gain a more thorough understanding of the role of serotonin in various aspects of social cognition (Young and Leyton, 2002). Accordingly, studies have experimentally manipulated 5-HT levels and examined the effect of increasing (or decreasing) central 5-HT levels on social cognition.

For instance, experimental depletion of tryptophan (TRP; precursor of 5-HT) in healthy volunteers made them judge couples as being less intimate and romantic (Bilderbeck et al., 2011). On the other hand, increasing 5-HT levels by means of TRP supplementation for 15 days decreased quarrelsome behavior in ‘quarrelsome’ men and women (Aan het Rot et al., 2006). Studies investigating the effects of experimental manipulation of 5-HT levels in humans suggest that lowering central 5-HT availability is associated with disruptive social behavior, whereas increasing 5-HT is associated with pro-social perception and behavior (Merens et al., 2007).

Manipulation of 5-HT levels also affects our response to unfairness as was shown in the ultimatum game (UG; Crockett et al., 2008, 2010). In the UG, the participant (responder) is exposed to offers to split a sum of money from other individuals. The responder can either accept the offer (in which the money is divided accordingly) or reject the offer (in which case both players receive nothing). The strategy maximizing gain is to accept every offer regardless of its fairness. However, very unfair offers (offering 20% of the total amount) have a 50% chance of being rejected (Güth et al., 1982; Bolton and Zwick, 1995), which indicates that emotion plays an important role in those decisions. Acute tryptophan depletion (ATD) was associated with a higher rejection rate (approximately 81%) of very unfair offers than sham ATD (∼65%) in healthy individuals (Crockett et al., 2008). This effect was independent of the size of the offer and ATD had no effect on self-reported mood or on response inhibition. In healthy participants, a single dose of the selective serotonin reuptake inhibitor (SSRI) citalopram (30 mg) was associated with a lower rejection rate of unfair offers (∼34%) than PLC and the norepinephrine reuptake inhibitor atomoxetine (60 mg; ∼48 and 50%, respectively; Crockett et al., 2010). This time the effect did not appear for very unfair offers but was restricted to moderately unfair offers (27–33% of the stake). Citalopram did not alter self-reported mood. In another study, healthy students who tended to reject unfair offers had lower platelet serotonin content than participants who tended to accept the offers (Emanuele et al., 2008). Finally, a PET study in 20 healthy males showed that individuals with low levels of 5-HT transporter binding in the dorsal raphe nucleus were more likely to reject unfair offers (Takahashi et al., 2012). In summary, UG behavior seems to be under serotonergic influence and the direction of the effect on the UG is consistent with the direction of the 5-HT manipulation (Crockett et al., 2008, 2010; Emanuele et al., 2008; Takahashi et al., 2012).

In contrast to acute serotonergic interventions, only a limited number of studies have investigated the effects of prolonged serotonergic interventions on social behavior in humans (Young and Leyton, 2002). Given (a) the finding that prolonged TRP intake in humans leads to prosocial behaviors (Aan het Rot et al., 2006) and (b) the relevance of prolonged serotonergic interventions in clinical settings (e.g., treatment of depression), the aim of the present study was to investigate the effects of a prolonged increase of 5-HT availability on social decision-making. We hypothesized that healthy volunteers who had taken TRP supplements for 6 days would accept more unfair offers than volunteers who had taken PLC capsules. We measured impulse control as a secondary outcome. We expected a selective effect on the UG.

## Materials and Methods

### Participants

The participants of this study are the same as reported in Cerit et al. (2013). Individuals were included who were healthy (self-report), non-smoker and whose grandparents were all West-European. The age range was 18–35 years and Body Mass Index was between 19 and 29 kg/m^2^. Exclusion criteria were a current diagnosis of depression or post-traumatic stress disorder, a lifetime history of psychosis, and use of medication, including oral contraceptives. Female participants were tested in their luteal phase of their menstrual cycle, defined as day 14–28 of their menstrual cycle. Written informed consent was obtained before data collection. The research was approved by the Medical Ethics Committee of Leiden University Medical Centre in The Netherlands. Participants received €40 upon completing the study.

### Instruments

#### Diagnosis

The Mini International Neuropsychiatric Interview (M.I.N.I.) was administered (Sheehan et al., 1997; Van Vliet et al., 2000) to assess psychiatric diagnoses.

#### Ultimatum Game

The UG consists of three conditions in which participants are exposed to fair (45% of stake), unfair (32% of stake), and most (i.e., very) unfair (21% of stake) offers from a “proposer,” who had split a sum of money provided by the experimenter. If the participant accepts the offer, both the proposer and participant will receive the money as proposed in the offer. In case of a rejection nobody receives money. Both fairness levels and offer size were manipulated. The value of the stake was either low (between 1 and 7 euro) or high (between 8 and 33 euro).

After 10 practice trials, participants were asked to respond to 48 offers (16 per fairness level). With each offer a photograph of a new proposer, the amount of the stake, and the amount of the offer was shown. The 48 photographs were counterbalanced for gender (24 male and 24 female proposers). All 48 offers were presented in a random order at each session. The participants were told that they would receive a percentage of the total amount that they had gained after having completed both sessions. In reality, there were no actual proposers and all participants received the same propositions. To increase credibility, the participants were first asked to split 24 sums of money (on paper) and had their photograph taken to be used in future experiments.

#### Impulsivity

The Go-Stop test is a stop-signal task and measures a dimension (impulse control) of impulsivity (Dougherty et al., 2005, 2010). In this task a series of 5-digit numbers are displayed for 500 ms with a 1,500 ms inter-stimulus interval. The 5-digit numbers appear in series, and some of these numbers are identical to the immediately preceding 5-digit number. Participants are instructed to respond to these matching numbers (Go Signal). Some of these matching numbers are first presented in black and then suddenly turn red. This is a Stop Signal cue, and the participants are instructed to withhold responding to any matching numbers that turn red. The timing of these stop signals varied across the testing session (e.g., 50, 150, 250, and 350 ms). The two dependent measures of interest were: (1) correct responses and (2) response inhibition failures. The primary dependent measure is the Go-Stop Ratio, which is the ratio of these two measures. The Go-Stop Ratio has been validated as a measure of the ability to inhibit an already initiated response, and data from the 150 ms stop delay typically provides the best group discrimination (Dougherty et al., 2010).

### Design and Procedure

This study had a randomized double-blind PLC-controlled design. Participants were randomly allocated to receive seven capsules containing either 400 mg TRP (total dose of 2.8 g/day) or PLC (cellulose microcrystalline) for a period of 6 days.

The dosage and duration were based on previous studies that had shown social-behavioral effects of TRP administration (3 g/day) after a period of 15 days (Aan het Rot et al., 2006) and cognitive effects after a single dose of 0.8 g TRP (Markus and Firk, 2009). The experimental procedure included two visits to the laboratory. The first visit to the laboratory included baseline measurements before the participants were provided with the capsules. The second visit included measurements after 6 days of intervention.

### First Visit to Laboratory (Pre-Intervention)

Upon arrival at the laboratory, participants provided written informed consent. Following the M.I.N.I. interview, participants completed the UG and Go-Stop test, respectively (as part of a larger test battery). At the end of the first visit, participants were provided with 42 capsules that contained 400 mg TRP or placebo (PLC). Oral and written instructions were provided regarding the timing of administration of capsules and lifestyle restrictions during the next 6 days and on the day of the second lab visit.

### Tryptophan Supplementation

Participants started to take the capsules the day after their first lab visit. They were instructed to take two capsules in the morning, two in the afternoon (before meals) and three in the evening (before 23.00 h). Participants received a diary in which they were asked to write down the exact time of intake and number of capsules. Compliance was not measured through blood sample analyses, however, participants were led to believe that compliance would be assessed at post-intervention through a saliva sample. Lifestyle instructions included: no smoking, no use of dietary supplements and vitamins, and consumption of alcohol limited to three units/day. Participants were also instructed to refrain from alcohol and caffeine-containing consumptions and avoid high carbohydrate meals on the day of their second visit. Further instructions for the day of the second visit included: no eating and drinking 1 h before arriving at the laboratory (except water), and no physical exercise at least 2 h before arrival. All test sessions started in the afternoon between noon and 5pm.

### Second Visit to Laboratory (Post-Intervention)

Upon arrival at the lab, participants handed in their diary regarding the intake of capsules. In addition, they were asked to fill out a debriefing questionnaire regarding compliance to the instructions during the previous 6 days. They were also interviewed about their compliance to the instructions for the second lab visit. Next, participants were asked to complete the UG and Go-Stop test in fixed order.

## Results

### Participant Characteristics

**FIGURE 1:**
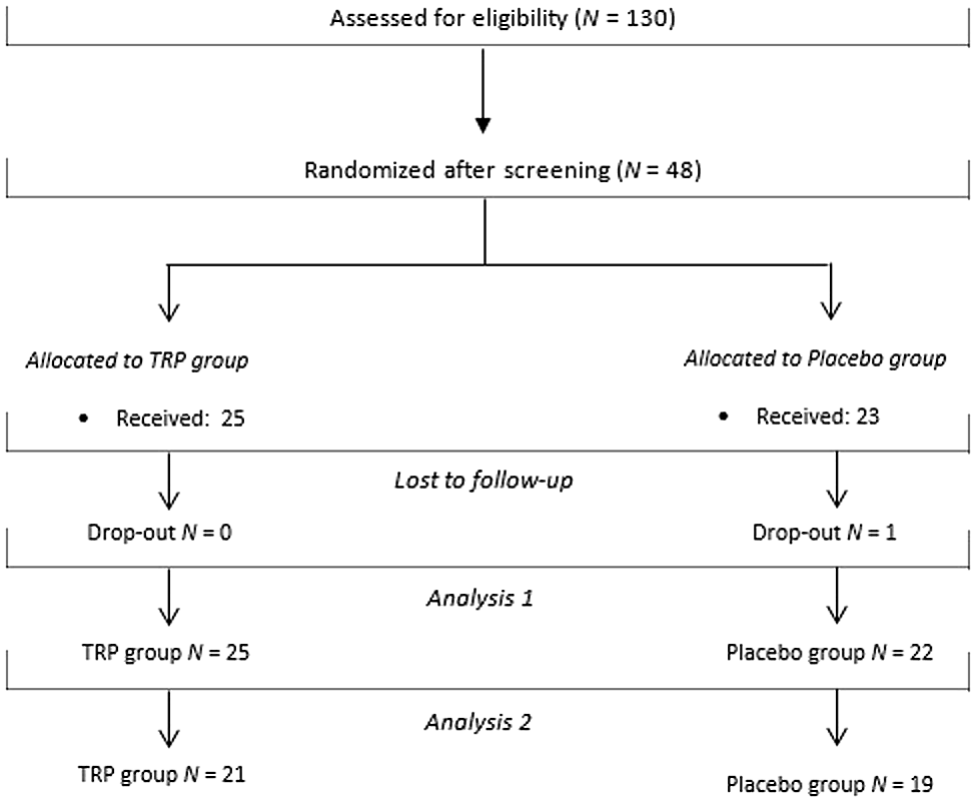
**Consort diagram**.

We contacted 184 individuals from a participant pool (*N* = 581) by email. Of these, 130 individuals expressed interest in the study (**Figure 1**). After screening for in- and exclusion criteria we included 48 participants. One participant dropped out of the study. The first two participants received TRP single-blind.

The demographic details of both groups are shown in **Table 1**. Groups did not differ significantly on demographic characteristics. One participant in the PLC group had a current diagnosis of panic disorder, and one participant in the TRP group had a specific phobia (for needles). No participant was on medication.

**Table 1:** Demographic characteristics of the tryptophan (TRP) and Placebo (PLC) groups^∗^.

	TRP (*N* = 25)	PLC (*N* = 22)
Age (M ± SD)	20.7 ± 3.6	19.9 ± 2.2
Female/male	14/11	9/13
Compliance (%)	97.6 ± 6.6	99.4 ± 1.8

*(M, Mean; SD, Standard Deviation); ^∗^In the PLC group one participant had a current diagnosis of panic disorder, and in the TRP group one participant had a specific phobia (for needles). Compliance % refers to the intake of the capsules over 6 days of intervention*.

The groups did not differ on UG behavior at baseline. A repeated measures ANOVA (RM-ANOVA) on UG rejection rates pre-intervention (baseline) with offer size (low, high) and fairness level (fair, unfair, and very unfair) as within subjects factors and intervention (TRP/PLC) as between subjects factors revealed that neither the main effect of Intervention nor any of the interactions with the factor Intervention were significant (all *F*s < 3.35, all *p*s > 0.074), Supplementary Figure S1.

### Compliance

According to self-report, 98% of the capsules were taken according to instructions. The minimum percentage of capsules taken by a participant was 69%. Three participants had taken two capsules in the morning of the second lab visit. All these participants were retained.

### Ultimatum Game

A RM-ANOVA on UG rejection rates post- intervention with offer size (low, high) and fairness level (fair, unfair, and very unfair) as within subjects factors and intervention (TRP/PLC) as between subjects factors revealed the expected main effect of fairness level [*F*(1.63,73.14) = 95.50, *p* < 0.001, $\eta_{p}^{2}$ = 0.680]; fair (*M* = 8.0), unfair (*M* = 40.7), very unfair (*M* = 68.2) and a main effect of offer size [*F*(1.00,45.00) = 5.87, *p* = 0.020, $\eta_{p}^{2}$ = 0.115]; low (*M* = 41.6) and high (*M* = 36.4). There was no main effect of Intervention [*F*(1.00,45.00) = 0.355, *p* = 0.554, $\eta_{p}^{2}$ = 0.008], nor a significant interaction (all *F*s < 1.87, all *p*s > 0.168).

This study was part of a larger study in which baseline measures pre-intervention were included. Repeated administration of behavioral measures, such as the UG, has the disadvantage of habituation and/or unwanted learning effects or changes in strategy. Therefore, an additional analysis was conducted in which participants who accepted all offers in the UG task (regardless of offer size or fairness level) on the second lab visit were excluded from the analyses. After exclusion of these seven participants (TRP, *N* = 4; PLC, *N* = 3) a RM-ANOVA on UG rejection rates post- intervention with Offer size (low, high) and Fairness level (fair, unfair, and very unfair) as within subjects factors and Intervention (TRP/PLC) as between subjects factors revealed main effect of Fairness level [*F*(1.93,73.34) = 157.76, *p* < 0.001, $\eta_{p}^{2}$ = 0.806] and Offer size [*F*(1.00,38.00) = 5.91, *p* = 0.020, $\eta_{p}^{2}$ = 0.135]. There was no main effect of Intervention [*F*(1.00,38.00) = 1.020, *p* = 0.319, $\eta_{p}^{2}$ = 0.026], nor a significant interaction between Offer size and Intervention [*F*(1.00,38.00) = 0.48, *p* = 0.508, $\eta_{p}^{2}$ = 0.012]. Importantly, however, the interaction between Fairness and Intervention was significant [*F*(1.93,73.34) = 3.715, *p* = 0.030, $\eta_{p}^{2}$ = 0.089] and remained significant (*p* = 0.015, $\eta_{p}^{2}$ = 0.127) when we corrected for possible pre-existing differences (i.e., entering UG rejection rates at the pre-intervention measurement as a covariates in the analyses)^1^.

**FIGURE 2:**
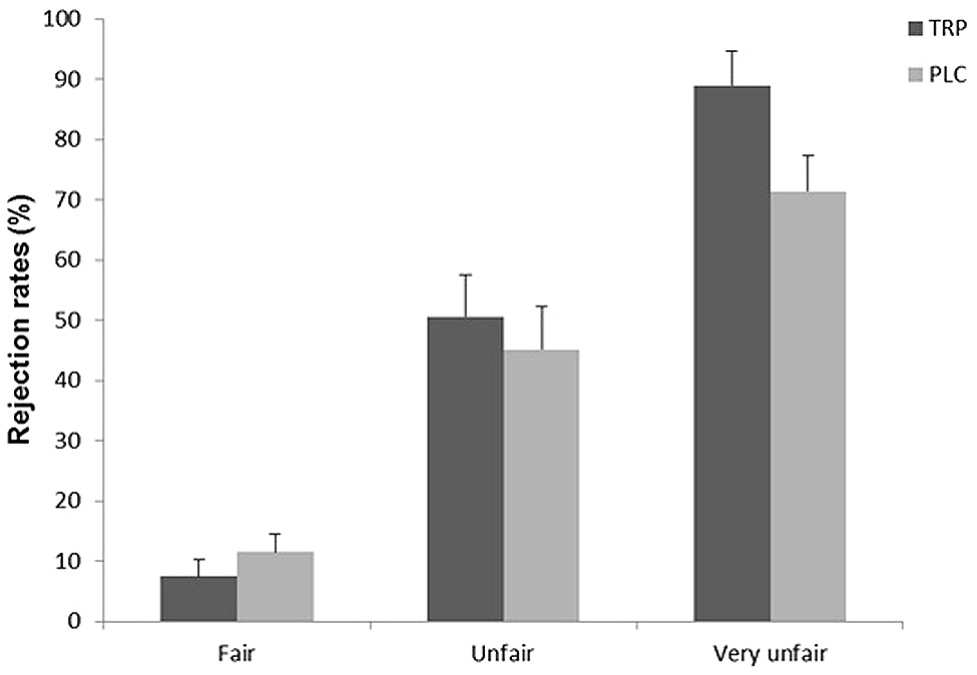
**Rejection rates (%) in the ultimatum game (UG) following TRP supplementation.** (TRP, Tryptophan; PLC, Placebo). Error bars represent SE.

Separate RM-ANOVAs with Offer size (low, high) as within subject factor and Intervention (TRP/PLC) as between subject factor were conducted on the rejection rate of the three levels of fairness post-intervention. The PLC and TRP groups did not differ on rejection rates of Fair and Unfair offers (both *F*s < 0.45, both *p*s > 0.50), however, there was a significant main effect of Intervention on rejection rates of Very Unfair offers [*F*(1,38) = 4.682, *p* = 0.037, $\eta_{p}^{2}$ = 0.110]. The TRP group rejected more very unfair offers compared to the PLC group (**Figure 2**; **Table 2**). There was no main or interaction effect involving offer size.

**Table 2:** Ultimatum Game (UG), rejection rates following 6 days of TRP/PLC (M ± SE).

Fairness	Offer size	Analysis 1		Analysis 2	
		TRP (*N*=25)	PLC (*N*=22)	TRP (*N*=21)	PLC (*N*=19)
Fair	Low	7 ± 3.05	12 ± 3.25	8 ± 3.50	14 ± 3.68
	High	6 ± 2.45	8 ± 2.61	7 ± 2.83	9 ± 2.98
Unfair	Low	47 ± 7.09	42 ± 7.56	55 ± 7.09	49 ± 7.46
	High	39 ± 7.23	36 ± 7.71	46 ± 7.70	41 ± 8.09
Very unfair	Low	76 ± 7.43	66 ± 7.92	90 ± 4.96^∗^	77 ± 5.22
	High	74 ± 8.33	57 ± 8.88	88 ± 7.14^∗^	66 ± 7.51

*TRP, Tryptophan; PLC, Placebo. Main effect of Intervention on rejection rates of very unfair offers [F287 (1,38) = 4.682, p = 0.037, $\eta_{p}^{2}$ = 0.110].*

### Impulsivity

**FIGURE 3:**
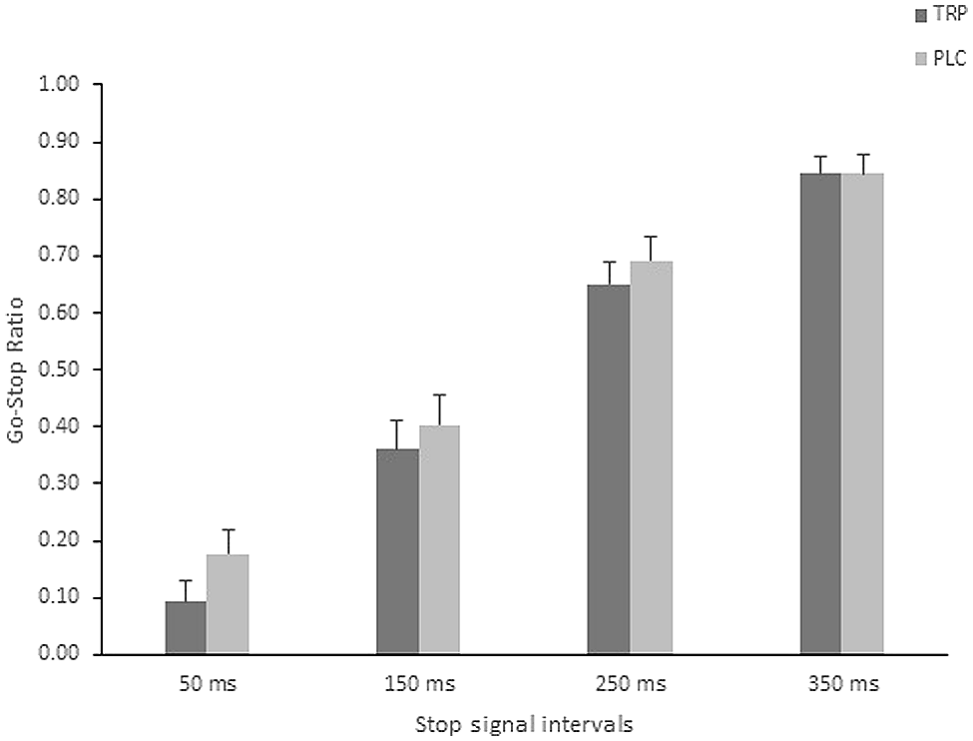
**Go-stop Ratio (stop signal task) following TRP Supplementation.** (TRP, Tryptophan; PLC, Placebo). Error bars represent SE.

A repeated measures ANOVA conducted on the primary dependent measure (i.e., Go-Stop ratio) of the impulsivity task post-intervention, with the four stop signal intervals (i.e., 50, 150, 250, and 350 ms) as within subject factors and Intervention (TRP/PLC) between subjects factors, revealed a main effect of stop signal intervals [*F*(2.68,120.39) = 269.90, *p* < 0.001, $\eta_{p}^{2}$ = 0.857]; 50 ms (*M* = 0.135), 150 ms (*M* = 0.382), 250 ms (*M* = 0.671), and 350 ms (0.844; **Figure 3**). There was no main effect of Intervention [*F*(1.00,45.00) = 0.738, *p* = 0.40, $\eta_{p}^{2}$ = 0.016], nor a significant interaction [*F* = 0.818, *p* = 0.48]. Moreover, the groups did not differ on impulsivity when seven participants were excluded, i.e., no interaction or main effect of intervention was found [*F*(1,38) = 0.873, *p* = 0.36].

## Discussion

The current study aimed at investigating effects of prolonged TRP supplementation on social decision-making as measured with the UG. The overall analysis showed that TRP supplementation had no significant effect on decision behavior, but the direction of effect was opposite from expectations. An additional analysis, in which seven participants who accepted all offers post-intervention were excluded, showed that the TRP-group rejected very unfair offers more often than the PLC group. TRP supplementation did not affect impulsivity, indicating that the effect was specific for social decision-making.

Our findings are not consistent with other studies in which healthy participants performed the UG following a serotonergic manipulation. A single dose of citalopram reduced the rejection rate of unfair offers compared to PLC condition in healthy volunteers (Crockett et al., 2010), whereas TRP depletion had an opposite effect (Crockett et al., 2008). Crucially, while previous studies mainly investigated acute effects of serotonin manipulations, we looked at prolonged TRP supplementation. Participants took high dosages of TRP for six consecutive days before the UG was administered again. The current results thus suggest that the effects of serotonin manipulation on social decision-making may importantly depend on the duration and intensity of the manipulation.

A possible explanation for our findings is that increasing serotonin availability during 6 days may make participants more sensitive to the socio-emotional aspect of the UG rather than to the monetary rewards. Elevated levels of serotonin may facilitate constructive social interactions by reducing aggression and increasing dominance (Young and Leyton, 2002). Dominance includes sociable behavior (i.e., prosocial actions; Kalma et al., 1993). Since fair treatment may be considered a premise for constructive social interactions, the TRP group may have become more sensitive to the social aspect of the task (i.e., being treated fairly by others).

Alternatively, since our participants did not take TRP on the day of testing, our findings might be due to the withdrawal of TRP that caused a relative depletion compared to the previous days. As we did not collect blood samples, we cannot be sure about the TRP concentrations at the time of testing. This relative depletion hypothesis, however, seems quite unlikely, considering that we found a lower cortisol response to social stress in the same participants, which is theoretically consistent with supplementation (Cerit et al., 2013).

Furthermore, previous studies have demonstrated that the effects of serotonin on cognitive functioning follow an inverted *U*-shape (see e.g., Chamberlain et al., 2006; Cano-Colino et al., 2014), explaining how baseline differences in neurochemical activity lead to divergent effects of drugs on cognitive performance. Acute and prolonged manipulation of serotonin may result in different baseline activities and thus be associated with different locations on the inverted *U*-shape. The aim of the current study was not to investigate whether the effect of TRP on social decision-making follows an inverted *U*-shape, however, this may be a possible explanation of contradictory findings from previous studies and the current, and may also be an important matter to consider for future investigations on the effect of increasing serotonin availability.

Our study shows that repeated administration of the UG may cause difficulties in interpreting the outcomes. Seven participants accepted all offers on the second test session (i.e., post-intervention). Examination of the health status, the demographical and compliance data of these seven participants did not indicate a difference that may explain their deviating behavior on the UG.

Accepting all offers is a gain maximizing strategy, which may simply reflect individual behavioral differences. Importantly, however, this strategy may obscure fairness considerations during social decision-making and thus lower the chance of finding effects of the intervention on this process of interest. Future studies should therefore try to avoid repeated administration of this paradigm.

One of the limitations of the current study is that our study sample included two participants with psychiatric conditions (i.e., specific phobia and panic disorder). Since the additional analysis conducted after excluding these two participants yielded similar results, it is highly unlikely that these two participants affected our main results.

Also, we have no information on the diet of the participants during the 6-day lasting TRP intervention, neither do we have information on the type of meal that participants have consumed on day 7. We did not take blood samples to measure peripheral parameters (e.g., TRP/LNAA ratios) which could have provided us with a manipulation check.

Future studies may benefit from measuring plasma amino acid levels, however, the usefulness is limited to a manipulation check as peripheral TRP concentrations are an indirect index of central serotonin.

Our study shows that increasing serotonin through prolonged TRP supplements increases the rejection of very unfair offers in healthy volunteers. If replicated, this implies that a prolonged increase of serotonin availability affects social decision-making differently than acute enhancement of serotonin availability. Given the interpretational difficulties of the UG, future studies may use other measures of social cognition and behavior, particularly with repeated test administration.

## Conflict of Interest Statement

The authors declare that the research was conducted in the absence of any commercial or financial relationships that could be construed as a potential conflict of interest.

## Footnotes

1 Please note that excluding the two participants with a psychiatric condition did not affect our outcomes. The interaction between Fairness and Intervention remained significant (*p* = 0.039). Follow-up analyses demonstrated increased rejection rates of the TRP group in response to very unfair offers (*p* = 0.043).

## References

[B1] Aan het Rot M. Moskowitz D. S. Pinard G. Young S. N. (2006). Social behaviour and mood in everyday life: the effects of tryptophan in quarrelsome individuals. *J. Psychiatry Neurosci.* 31 253–262. 16862243 PMC1488902

[B2] Bilderbeck A. C. McCabe C. Wakeley J. McGlone F. Harris T. Cowen P. J. (2011). Serotonergic activity influences the cognitive appraisal of close intimate relationships in healthy adults. *Biol. Psychiatry* 69 720–725. 10.1016/j.biopsych.2010.12.038 21396628

[B3] Bolton G. E. Zwick R. (1995). Anonymity versus punishment in ultimatum bargaining. *Games Econ. Behav.* 10 95–121. 10.1006/game.1995.1026

[B4] Brown G. L. Goodwin F. K. Ballenger J. C. Goyer P. F. Major L. F. (1979). Aggression in humans correlates with cerebrospinal fluid amine metabolites. *Psychiatry Res.* 1 131–139. 10.1016/0165-1781(79)90053-2 95232

[B5] Cano-Colino M. Almeida R. Gomez-Cabrero D. Artigas F. Compte A. (2014). Serotonin regulates performance nonmonotonically in a spatial working memory network. *Cereb. Cortex* 24 2449–2463. 10.1093/cercor/bht096 23629582

[B6] Cerit H. Jans L. A. Van der Does A. J. W. (2013). The effect of tryptophan on the cortisol response to social stress is modulated by the 5- HTTLPR. *Psychoneuroendocrinology* 38 201–208. 10.1016/j.psyneuen.2012.05.016 22717170

[B7] Chamberlain S. R. Müller U. Robbins T. W. Sahakian B. J. (2006). Neuropharmacological modulation of cognition. *Curr. Opin. Neurol.* 19 607–612. 10.1097/01.wco.0000247613.28859.77 17102701

[B8] Crockett M. J. Clark L. Hauser M. D. Robbins T. W. (2010). Serotonin selectively influences moral judgment and behavior through effects on harm aversion. *Proc. Natl. Acad. Sci. U.S.A.* 107 17433–17438. 10.1073/pnas.1009396107 20876101 PMC2951447

[B9] Crockett M. J. Clark L. Tabibnia G. Lieberman M. D. Robbins T. W. (2008). Serotonin modulates behavioral reactions to unfairness. *Science* 320 1739–1739. 10.1126/science.1155577 18535210 PMC2504725

[B10] Dougherty D. Mathias C. Marsh D. Jagar A. (2005). Laboratory behavioral measures of impulsivity. *Behav. Res. Methods* 37 82–90. 10.3758/bf03206401 16097347

[B11] Dougherty D. M. Richard D. M. James L. M. Mathias C. W. (2010). Effects of acute tryptophan depletion on three different types of behavioral impulsivity. *Int. J. Tryptophan Res.* 3 99–111. 10.4137/ijtr.s4317 22084592 PMC3195237

[B12] Emanuele E. Brondino N. Bertona M. Re S. Geroldi D. (2008). Relationship between platelet serotonin content and rejections of unfair offers in the ultimatum game. *Neurosci. Lett.* 437 158–161. 10.1016/j.neulet.2008.04.006 18448251

[B13] Güth W. Schmittberger R. Schwartze B. (1982). An experimental analysis of ultimatum bargaining. *J. Econ. Behav. Organ.* 3 367–388. 10.1016/0167-2681(82)90011-7

[B14] Kalma A. P. Visser L. Peeters A. (1993). Sociable and aggressive dominance: personality differences in leadership style? *Leadersh. Q.* 4 45–64. 10.1016/1048-9843(93)90003-c

[B15] Markus C. R. Firk C. (2009). Differential effects of tri-allelic 5- HTTLPR polymorphisms in healthy subjects on mood and stress performance after tryptophan challenge. *Neuropsychopharmacology* 34 2667–2674. 10.1038/npp.2009.92 19675533

[B16] Merens W. Van der Does A. W. Spinhoven P. (2007). The effects of serotonin manipulations on emotional information processing and mood. *J. Affect. Disord.* 103 43–62. 10.1016/j.jad.2007.01.032 17363069

[B17] Owens M. J. Nemeroff C. B. (1994). Role of serotonin in the pathophysiology of depression: focus on the serotonin transporter. *Clin. Chem.* 40 288–295. 7508830

[B18] Sandi C. Haller J. (2015). Stress and the social brain: behavioural effects and neurobiological mechanisms. *Nat. Rev. Neurosci.* 16 290–304. 10.1038/nrn3918 25891510

[B19] Sheehan D. V. Lecrubier Y. Harnett Sheehan K. Janavs J. Weiller E. Keskiner A. (1997). The validity of the Mini International Neuropsychiatric Interview (MINI) according to the SCID-P and its reliability. *Eur. Psychiatry* 12 232–241. 10.1016/S0924-9338(97)83297-X

[B20] Takahashi H. Takano H. Camerer C. F. Ideno T. Okubo S. Matsui H. (2012). Honesty mediates the relationship between serotonin and reaction to unfairness. *Proc. Natl. Acad. Sci. U.S.A.* 109 4281–4284. 10.1073/pnas.1118687109 22371595 PMC3306703

[B21] Van Vliet I. M. Leroy H. Van Megen H. J. M. (2000). *De MINI-Internationaal Neuropsychiatrisch Interview.* Leiden: Leiden University Medical Center.

[B22] Young S. N. Leyton M. (2002). The role of serotonin in human mood and social interaction: insight from altered tryptophan levels. *Pharmacol. Biochem. Behav.* 71 857–865. 10.1016/S0091-3057(01)00670-0 11888576

