# Supplementary material for: Tryptophan supplementation and the response to unfairness in healthy volunteers
Source: Front Psychol. 2015 Jul 16;6:1012. doi: 10.3389/fpsyg.2015.01012 (PMC4503888; doi:10.3389/fpsyg.2015.01012)
Supplement: Supplementary file 1 [file Data_Sheet_1.DOCX]

**FIGURE S1**

**Rejection rates (%) in the Ultimatum Game at baseline**

(TRP; Tryptophan, PLC; Placebo). Error bars represent Standard Error (SE). There were no significant effects involving intervention (all Fs < 3.35, all *p*s > 0.074)

**FIGURE S2a**

**Overall acceptance rates after one week of (PLC or TRP) intervention**

(TRP; Tryptophan, PLC; Placebo)


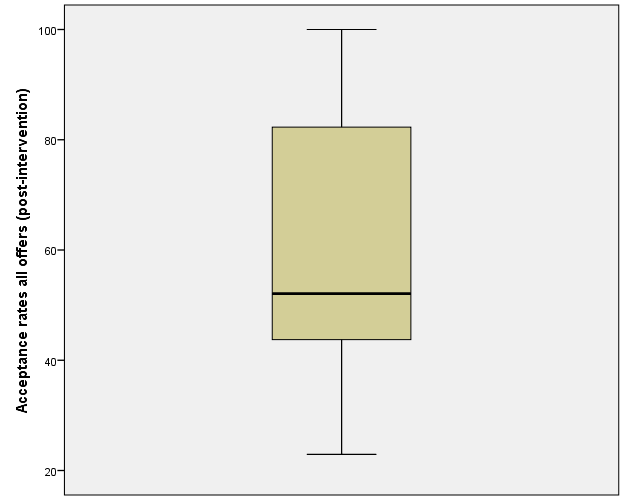


**FIGURE S2b**

**“Very Unfair Offers” acceptance rates after one week of (PLC or TRP) intervention**

(TRP; Tryptophan, PLC; Placebo)


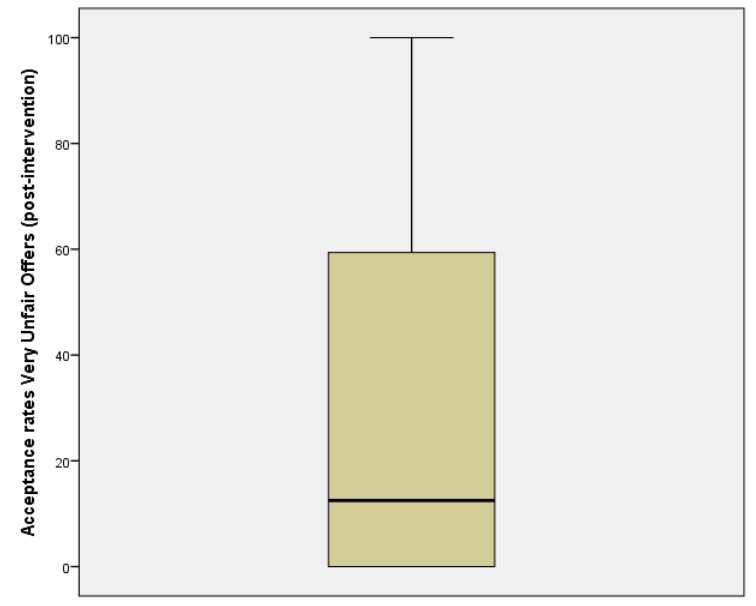


**FIGURE S3**

**Overall rejection rates after one week of (PLC or TRP) intervention**
